# Supplementary material for: Values, motivation, and physical activity among Chinese sport sciences students
Source: PLoS One. 2025 Feb 24;20(2):e0316731. doi: 10.1371/journal.pone.0316731 (PMC11849836; doi:10.1371/journal.pone.0316731)
Supplement: S1 File — (DOCX) [file pone.0316731.s001.docx]

**Supporting Information**

**Materials and Method**

**Participants**

Three hundred and seventy-nine sports science college students participated in the present study. Two hundred and forty-seven participants made no sporting competition (*M*_age_=2.67, *SD*=1.32; *M*_BMI_=22.52, *SD*=7.44), while 132 participants made sporting competition (*M*_age_=2.36, *SD*=1.06; *M*_BMI_ =21.64, *SD*=2.93), 83 participants made competition at a regional level (*M*_age_=2.35, *SD*=.99; *M*_BMI_=21.90, *SD*=2.40), 42 participants at a national level (*M*_age_=2.31, *SD*=1.12; *M*_BMI_ =21.05, *SD*=3.84), and 7 participants at an international level (*M*_age_=2.86, *SD*=1.46; *M*_BMI_=22.02, *SD*=1.94).

**Measures**

**Physical Activity Behavior.** Physical activity was assessed with the Chinese version of the International Physical Activity Questionnaire short version (IPAQ-short) [1]. The IPAQ short version assesses physical activity undertaken across a comprehensive set of domains in daily life through 7 items and three different intensities in global physical activity. For example, one question for measuring vigorous physical activity is, “During the last 7 days, how many days did you do vigorous physical activities like heavy lifting, digging, aerobics, or fast bicycling?”. Participants answer the frequency in the last 7 days and further answer the time they spend on one of those days.

The results can be described as a continuous score and reported as median MET-minutes. Median values can be computed for walking (W), moderate-intensity activities (M), and vigorous-intensity activities (V) using the following formulas: walking MET-minutes/week=3.3*walking minutes*walking days; moderate MET-minutes/week=4.0*moderate-intensity activity minutes*days; vigorous MET-minutes/week=8.0*vigorous-intensity activity minutes*days. A combined total physical activity MET-min/week can be computed as the sum of walking + Moderate + Vigorous MET-min/week scores.

**Personal Values.** Participants completed the Chinese version of the Portrait Values Questionnaire (PVQ-21) [2]. The PVQ-21 measures the 10 original categories of value types with two or three items by category. PVQ-21 tends to be more valid for the four higher-order values [3]. Each question refers to a portrait of an individual that participants are asked to identify with by responding from 1 (not at all like me) to 6 (very much like me). For example, an item that measures the importance participants attached to self-direction is: “Thinking up new ideas and being creative. He likes to do things in his original way.” The 21 items can be combined into 4 higher-order values or 10 broad values.

**Motivation to physical activity.** Participants completed the Chinese version of the Behavioral Regulations in Exercise Questionnaire 2 (BREQ-2) [4] (see in Table S1).

**Table S1. Definition and examples of separate motivational regulation in BREQ-2.**

| Types of behavioral regulation | Definition | Examples |
| --- | --- | --- |
| Intrinsic regulation | Motivation due to the inherent enjoyment derived from the behavior itself | “I exercise because it is fun and pleasurable” |
| Integrated regulation | Motivation to engage in behaviors that are in congruence with other central personal goals and values | “I exercise because I consider exercise a fundamental part of who I am” |
| Identified regulation | Motivation reflects the personal value of the behavior’s outcomes. | I exercise because I value the benefits of exercising |
| Introjected regulation | Motivation reflecting internal pressures such as contingent self-worth, guilt, shame, and need for external approval | I exercise because I will feel guilty when I don’t |
| External regulation | Motivation to comply with external pressure or rewards. | I exercise because my physician says I should |
| Amotivation | The state of lacking intention to act. | I can’t see why I should bother exercising |

Specifically, we used in the present study autonomous (the average of the identified regulation and intrinsic regulation, *r*=.61, *p*<.001) and controlled motivation (the average of external regulation and introjected regulation, *r*=.10, *p*=.054). Each item describes the motivation for exercise that participants are asked to identify with by responding from 0 (not true for me) to 4 (Very true for me). For example, one item that measures internal regulations is “I exercise because other people say I should”. The behavioral regulation could combine into five separate behavioral regulations, one autonomous and one controlled motivation, or one composite relative autonomous motivation score.

**Data Analysis**

Descriptive analysis and correlation analysis of global physical activity and higher-order values were tested by mixed-model Analysis of Variance (ANOVA) and Pearson bivariate analysis. Furthermore, simple linear regression analyses were employed to investigate the predictive value on global physical activity for gender (*B*=.05, *p*=.407, 95%CI [-.06, .15], with females coded as -.5 and males as +.5), age (*B*=-.11, *p*=.037, 95%CI [-.21, -.01]), Body Mass Index (BMI, calculated as weight in kilograms divided by the square of height in meters, *B*=.11, *p*=.043, 95%CI [.00, .21]), and competition level (coded as 1 for “no competition”, 2 for “local level”, 3 for “regional level”, 4 for “national level”, and 5 for “international level”, *B*=.10, *p* =.057, 95%CI [-.00, .20]).

To evaluate the model’s fit, we used the Comparative Fit Index (CFI) and the Root Mean Square Error of Approximation (RMSEA), adopting thresholds of CFI ≥ .90 and RMSEA ≤ .08, respectively, in conjunction with a *χ²*/*df* ratio of less than 5, as benchmarks for acceptable model fit, following the criteria established by Hu and Bentler (1999) [5]. Following Hayes’s bootstrapping method [6], to consider a mediator has mediational effect when (1) the indirect effect (IE) of four higher order values (Self-transcendence, Self-enhancement, Openness to change and Conservation) on global physical activity through motivation (relative autonomous index, autonomous motivation, controlled motivation, amotivation) (i.e., IE=path a * path b; a=the effect of four higher order values on the mediator of motivation, b=the effect of motivation on physical activity), and (2) the bias corrected 95% CT around the IE from 5000 bootstrap re-sample. We accept the IE as statistically significant only if its bias corrected 95% CI excluded zero.

**Results**

**Reliability and Validity**

The reliability and validity of the Personal Values Questionnaire (PVQ) and the Behavioral Regulation in Exercise Questionnaire-2 (BREQ-2) were assessed by Cronbach’s alpha and Confirmatory Factor Analysis in SPSS software (Version 21.0; IBM) [7].

The PVQ-21 measures the four higher-order values with each through three or four items: openness to change (α=.710), self-transcendence (α=.665), conservation (α=.594), self-enhancement (α=.585). Also, the measurement models for self-transcendence (*χ*^2^/*df*=1.584, CFI=.957, IFI=.958, RMSEA=.097), self-enhancement (*χ*^2^/*df*=4.583, CFI=.957, IFI=.958, RMSEA=.114), openness to change (*χ*^2^/*df*=7.681, CFI=.846, IFI=.848, RMSEA=1.830) and Conservation (*χ*^2^/*df*=2.557, CFI=.995, IFI=.995, RMSEA=.064) were acceptable according to model fit index.

The BREQ-2 measures the five behavioral regulations with each through three or four items: amotivation (α=.844), external motivation (α=.779), introjected motivation (α=.699), identified motivation (α=.685), intrinsic motivation (α=.808). The BREQ-2 presented acceptable reliability and validity (*χ*^2^/*df*=3.810, CFI=.871, IFI=.872, RMSEA=.086).

**Descriptive statistics**

**Global physical activity in Chinese.** Global physical activity was analyzed with a 2 x 2 between-subject ANOVA, with Gender (Male vs. Female) and Competition statement (Competition vs. leisure) as factors (See in Table S2).

**Table S2. Descriptive analysis and Cronbach α in research variables.**

|  | Global (N=379) | | Male (N=280) | | Female (N=99) | | Competition (N=132) | | Leisure (N=247) | |
| --- | --- | --- | --- | --- | --- | --- | --- | --- | --- | --- |
|  | Mean (SD) | 95%CI | Mean (SD) | 95%CI | Mean (SD) | 95%CI | Mean (SD) | 95%CI | Mean (SD) | 95%CI |
| GPA | 698.96 (4245.33) | [6532.08, 7418.14] | 7012.28 (4178.75) | [6502.64, 7479.39] | 6892.40 (4449.93) | [6004.39, 7736.02] | 7593.73 (4271.18) | [6839.26, 8343.09] | 6653.50 (4203.54) | [6137.04, 7151.47] |
| Openness (r) | 3.54(.72) | [3.47, 3.61] | 3.51 (.71) | [3.43, 3.59] | 3.63 (.75) | [3.48, 3.78] | 3.56 (.69) | [3.44, 3.67] | 3.54 (.74) | [3.45, 3.64] |
| Self-T(r) | 3.57(.69) | [3.50, 3.64] | 3.58 (.73) | [3.50, 3.67] | 3.55 (.60) | [3.44, 3.66] | 3.44 (.72) | [3.32, 3.56] | 3.65 (.68) | [3.56, 3.73] |
| Conser (r) | 3.37(.66) | [3.31, 3.44] | 3.45 (.64) | [3.37, 3.52] | 3.18 (.70) | [3.04, 3.31] | 3.30 (.68) | [3.18, 3.42] | 3.42 (.65) | [3.34, 3.50] |
| Self-E (r) | 3.38(.75) | [3.31, 3.46] | 3.42 (.73) | [3.32, 3.50] | 3.29 (.82) | [3.12, 3.44] | 3.39 (.76) | [3.26, 3.53] | 3.38 (.76) | [3.28, 3.47] |
| Openness (c) | .06 (.46) | [.02, .11] | .01 (.45) | [-.04, .07] | .20 (.46) | [.11, .29] | .12 (.44) | [.04, .20] | .03 (.47) | [-.03, .09] |
| Self-T (c) | .09 (.43) | [.05, .13] | .08 (.45) | [.03, .14] | .11 (.42) | [.03, .20] | .01 (.51) | [-.08, .10] | .13 (.39) | [.09, .18] |
| Conser (c) | -.11 (.43) | [-.15, -.06] | -.05 (.40) | [-.11, -.01] | -.25 (.50) | [-.35, -.15] | -.13 (.48) | [-.22, -.05] | -.09 (.41) | [-.14, -.04] |
| Self-E (c) | -.10 (.56) | [-.16, -.05] | -.08 (0。56) | [-.15, -.02] | -.15 (.56) | [-.26, -.04] | -.03 (.56) | [-.13, .06] | -.13 (.56) | [-.20, -.06] |
| Amotivation | .50(.82) | [.42, .59] | .49 (.85) | [.40, .60] | .53 (.74) | [.40, .69] | .58 (.86) | [.44, .73] | .46 (.80) | [.36, .57] |
| External | .77(.91) | [.68, .87] | .76 (.92) | [.65, .87] | .83 (.89) | [.67, 1.01] | .78 (.95) | [.63, .97] | .77 (.89) | [.66, .89] |
| Introjected | 1.83(1.08) | [1.71, 1.93] | 1.93 (1.09) | [1.79, 2.04] | 1.56 (1.02) | [1.36, 1.77] | 1.88 (1.18) | [1.67, 2.07] | 1.81 (1.03) | [1.68, 1.93] |
| Identified | 2.79(.82) | [2.71, 2.88] | 2.89 (.81) | [2.79, 2.98] | 2.52 (.80) | [2.36, 2.68] | 2.68 (.89) | [2.52, 2.83] | 2.85 (.78) | [2.75, 2.95] |
| Intrinsic | 3.27(.80) | [3.19, 3.35] | 3.39 (.71) | [3.31, 3.48] | 2.93 (-.00) | [2.73, 3.11] | 3.06 (.93) | [2.90, 3.21] | [3.29, 3.47] | [3.29, 3.47] |
| Autonomous | 6.07 (1.46) | [5.91, 6.21] | 6.28 (1.36) | [6.11, 6.44] | 5.45 (1.56) | [5.13, 5.76] | 5.74 (1.65) | [5.47, 6.03] | 6.24 (1.32) | [6.07, 6.39] |
| Controlled | 2.61 (1.48) | [2.46, 2.75] | 2.69 (1.50) | [2.51, 2.86] | 2.39 (.00) | [2.10, 2.68] | 2.67 (1.56) | [2.39, 2.94] | 2.58 (1.44) | [2.39, 2.76] |
| RAI | 1.51(6.21) | [9.90, 11.17] | 11.05 (6.11) | [1.26, 11.74] | 9.01 (6.28) | [7.71, 1.24] | 9.36 (6.62) | [8.21, 1.49] | 11.13 (5.91) | [1.31, 11.83] |

*Note*. GPA=global physical activity, Self-T (r)=Self-transcendence raw data, Openness (r)=Openness to change raw data, Self-E (r)=Self-enhancement raw data, Conser (r)=Conservation raw data, Self-T (c)=Self-transcendence centered data, Openness (c)=Openness to change centered data, Self-E (c)=Self-enhancement raw data, Conser (c)=Conservation centered data, External=external regulation, Introjected=introjected regulation, Identified=identified regulation, Intrinsic=Intrinsic regulation, Autonomous=Autonomous motivation, Controlled=controlled motivation, RAI=relative autonomous index.

Results indicated only a marginally significant main effect of competition level, *F*(1, 375)=3.69, *p*=.056, ŋ²=.01, *R*²=.004, The marginally significant main effect of competition level suggested that participants doing competition level (*M*=7593.72, *SD*=4271.77) were more physically active than participants not doing physical activity in competition (*M*=6653.49, *SD*=4203.54).

**Four higher domains of values in Chinese participants**. The importance of the four higher category of values was analyzed with a 2 x 2 X 4 (Gender X Competition level X higher order values) mixed ANOVA, with Gender (Male vs. Female) and Competition level (Competition level vs. leisure) as between-subjects factor and Higher order values (self-transcendence vs. self-enhancement vs. openness to change vs. conservation) as within-subjects factor (See in Table S2).

Results indicated a significant main effect of higher order value type, *F*(3,1125)=17.51, *p*<.001, η^2^=.04, a significant two-way interaction between higher order type and gender, *F*(3,1125)=5.74, *p*=001, η^2^=.016, and a significant interaction between higher order value type and competition level, *F*(3,1125)=3.01, *p*=.029, η^2^=.01.

The significant main effect of higher order values indicates that participants attached more importance to self-transcendence values (*M*=.09, *SD*=.43) and openness to change values (*M*=.06, *SD*=.43) than self-enhancement values (*M*=-.10, *SD*=.43) and conservatism values (*M*=-.11, *SD*=.43; *ps*<.001). Nevertheless, they attached the same level of importance to self-transcendence values as openness to change values (*p*=.481) and the same level of importance to self-enhancement values as conservation values (*p*=.876).

The interaction between gender and higher order values indicates that male participants attached more importance to self-transcendence values (*M*=.08, *SD*=.44) than self-enhancement values (*M*=-.08, *SD*=.56; *p*=.043) and a marginally greater importance to self-transcendence values (*M*=.08, *SD*=.44) than conservation values (*M*=-.05, *SD*=.39; *p*=.078). Nevertheless, they attached the same level of importance to self-transcendence (*M*=.08, *SD*=.44) values as openness to change values (*M*=.01, *SD*=.45; *p*=.283) and the same level of importance to self-enhancement (*M*=-.08, *SD*=.56; *p*=.043) values as conservation values (*M*=-.05, *SD*=.39; *p*=.645).

The interaction between gender and higher order values indicates that female participants attached more importance to openness values (*M*=.19, *SD*=.46) and self-transcendence values (*M*=.11, *SD*=.41) than self-enhancement values (*M*=-.14, *SD*=.55; *p*=.043) and conservation values (*M*=-.25, *SD*=.50; *ps*<.001). Nevertheless, they attached the same level of importance to openness to change values (*M*=.19, *SD*=.46) as self-transcendence values (*M*=.11, *SD*=.41; *p*=.175), while they attached more importance to self-enhancement (*M*=-.15, *SD*=.55) than conservation values (*M*=-.25, *SD*=.50; *p*<.001).

The interaction between gender and higher order values also indicate that female participants attach more importance to openness to change values (*M*=.19, *SD*=.46 vs *M*=.01, *SD*=.45; *p*<.001) and marginally more importance to self-transcendence values than male participants (*M*=.11, *SD*=.41 vs *M*=.08, *SD*=.44; *p*=.071). Male participants attached more importance to self-enhancement values (*M*=-.08, *SD*=.56 vs *M*=-.14, *SD*=.55; *p*<.001) and more importance to conservation values than female participants (*M*=-.05, *SD*=.39 vs *M*=-.25, *SD*=.50; *p*<.001).

The interaction between competition level and higher order values indicates that participants doing competition attached more importance to openness values (*M*=.12, *SD*=.44) than self-enhancement (*M*=-.03, *SD*=.55; *p*=.027) and conservation values (*M*=-.13, *SD*=.47; *p*<.001). Nevertheless, they attached as importance to openness values (*M*=.12, *SD*=.44) as self-transcendence values (*M*=.00, *SD*=.51; *p*=.103) and the same level of importance to self-enhancement (*M*=-.03, *SD*=.55) values as conservation values (*M*=-.13, *SD*=.47; *p*=.228).

The interaction between competition level and higher order values indicates that participants doing physical activity only in leisure time attached more importance to self-transcendence values (*M*=.13, *SD*=.38) than conservation values (*M*=-.09, *SD*=.41; *p*=.001) and self-enhancement values (*M*=-.13, *SD*=.56; *p*<.001). Nevertheless, they attached as importance to self-transcendence values (*M*=.13, *SD*=.38) as openness values (*M*=.02, *SD*=.46; *p*=.163) and the same level of importance to self-enhancement values (*M*=-.13, *SD*=.56) as conservation values (*M*=-.09, *SD*=.41; *p*=.754).

Finally, the interaction between competition level and higher order values indicates that participants doing physical activity in competition attached more importance to openness values (*M*=.12, *SD*=.44 vs *M*=.02, *SD*=.47; *p*<.001) and self-enhancement values (*M*=-.03, *SD*=.55 vs *M*=-.13, *SD*=.56; *p*<.001) than participants doing physical activity only in leisure. Participants doing physical activity in leisure attached more importance to self-transcendence values (*M*=.13, *SD*=.38 vs *M*=.00, *SD*=.51; *p*<.001) and more importance to conservation values (*M*=-.09, *SD*=.41 vs *M*=-.13, *SD*=.47; *p*=.007) than participants doing physical activity in competition.

**Autonomous motivation**

The motivation was analyzed with a 2 X 2 X 5 (Gender X Competition level X Motivation) mixed ANOVA, with Gender (Male vs. Female) and Competition level (Competition level vs. leisure) as between-subjects factor and Motivation (Amotivation, External motivation, Introjected motivation, Identified motivation and Intrinsic motivation) as within-subjects factor (See in Table S2).

Results indicated a significant main effect of motivation, *F*(4,1500)=522.67, *p*<.001, η^2^=.58, a significant two-way interaction between motivation and gender, *F*(4,1500)=5.93, *p*<.001, η^2^=.02, and a significant interaction between motivation and competition level, *F*(5,1500)=3.42, *p*=.008, η^2^=.01.

The significant main effect of motivation indicated that intrinsic motivation were more important than identified motivation, which in turn were more important than introjected motivation. Also, introjected motivation was more important than external motivation, which in turn was more important than amotivation.

The significant interaction between gender and motivation indicated mainly that male participants had a higher intrinsic motivation, identified motivation, introjected motivation than female participants, while there were no differences according to gender for amotivation and external motivation. The significant interaction between competition level and motivation indicated mainly that intrinsic motivation was higher for participants not making competition that for participants who made competition (see in Table S2).

**Correlation analysis**

Bivariate correlation analysis results indicate that the centered score of Openness to change was positively correlated to global physical activity (*r*=.11, *p*=.035) and the centered score of conservation value was negatively correlated to global physical activity (*r*=-.11, *p*=.035).

Moreover, centered score of self-enhancement value was positively correlated to controlled motivation (*r*=.22, *p*<.001), external regulation (*r*=.13, *p*=.014), introjected regulation (*r*=.19, *p*<.001) and was negatively correlated to intrinsic regulation (*r*=-.10, *p*=.048).

The centered score of openness to change was positively correlated to intrinsic regulation (*r*=.11, *p*=.033). The centered score of conservation was negatively correlated to autonomous motivation (*r*=-.12, *p*=.019), and was negatively correlated to intrinsic regulation (*r*=-.13, *p*=.015).

At last, global physical activity was positively correlated to autonomous motivation (*r*-.12, *p*=.018) and identified regulation (*r*=.12, *p*=.020). Conversely, global physical activity was negatively correlated to amotivation (*r*=-.16, *p*=.002).

**Table S3. Interrelations between higher-order values, motivation, global physical activity, age, BMI, and competition level.**

|  | **Mean** | **SD** | **95% CI** | **2** | **3** | **4** | **5** | **6** | **7** | **8** | **9** | **10** | **11** | **12** | **13** | **14** | **15** |
| --- | --- | --- | --- | --- | --- | --- | --- | --- | --- | --- | --- | --- | --- | --- | --- | --- | --- |
| **1.age** | 2.56 | 1.24 | [2.44, 2.69] | .06 | -.09^+^ | -.10^*^ | .10^+^ | -.05 | -.06 | .10^+^ | .06 | .08 | .09^+^ | .11^*^ | .02 | .05 | .06 |
| **2.BMI** | 22.21 | 6.26 | [21.75, 22.91] | - | -.07 | .10^+^ | -.06 | .06 | -.03 | -.01 | -.01 | -.02 | -.01 | .02 | -.04 | -.06 | .05 |
| **3.level** | 1.50 | .76 | [1.42, 1.57] |  | - | .09^+^ | -.08 | .05 | .09^+^ | -.04 | -.19^**^ | .04 | .04 | .04 | .02 | -.09^+^ | -.25^**^ |
| **4.GPA** | 698.96 | 4245.34 | [6568.31, 7379.45] |  |  | - | -.08 | .00 | .11* | -.11^*^ | .12^*^ | -.05 | -.16^**^ | -.06 | -.02 | .12* | .10^+^ |
| **5.Self-T** | .09 | .44 | [.04, .13] |  |  |  | - | -.41^**^ | -.33^**^ | .03 | .05 | -.03 | -.01 | -.06 | .02 | .04 | .06 |
| **6.Self-E** | -.10 | .56 | [-.16, -.05] |  |  |  |  | - | -.25^**^ | -.26^**^ | -.05 | .22^**^ | .00 | .13* | .19^**^ | .02 | -.10* |
| **7.Openn** | .06 | .46 | [-.01, .122] |  |  |  |  |  | - | -.62^**^ | .08 | -.06 | -.03 | -.02 | -.06 | .04 | .11^*^ |
| **8.Conser** | -.11 | .44 | [-.15, -.06] |  |  |  |  |  |  | - | -.12* | -.05 | .10^+^ | .01 | -.07 | -.09^+^ | -.13^*^ |
| **9.Autonomous** | 6.07 | 1.46 | [5.93, 6.21] |  |  |  |  |  |  |  | - | .14^**^ | -.39^**^ | -.25^**^ | .40^**^ | .90^**^ | .90^**^ |
| **10.Controlled** | 2.61 | 1.48 | [2.47, 2.76] |  |  |  |  |  |  |  |  | - | .45^**^ | .69^**^ | .79^**^ | .30^**^ | -.06 |
| **11.Amotivation** | .50 | .82 | [.42, .58] |  |  |  |  |  |  |  |  |  | - | .67^**^ | .05 | -.29^**^ | -.41^**^ |
| **12.External** | .78 | .91 | [.67, .86] |  |  |  |  |  |  |  |  |  |  | - | .10^+^ | -.13^**^ | -.33^**^ |
| **13.Introjected** | 1.83 | 1.08 | [1.73, 1.95] |  |  |  |  |  |  |  |  |  |  |  | - | .52^**^ | .20^**^ |
| **14.Identified** | 2.79 | .82 | [2.72, 2.88] |  |  |  |  |  |  |  |  |  |  |  |  | - | .61^**^ |
| **15.Intrinsic** | 3.27 | .80 | [3.19, 3.36] |  |  |  |  |  |  |  |  |  |  |  |  |  | - |

*Notes:* + means *p*<.01, * means *p*<.05, ** means *p*<.01, Self-T=Self-Transcendence, Self-E=Self-enhancement, Openness=Openness to change, Conser=Conservation, Autonomous=Autonomous Motivation, Controlled=Controlled Motivation, External=External Regulation, Introjected=Introjected Regulation, Identified =Identified Regulation, Intrinsic=Intrinsic Regulation.

**Mediation analysis**

In order to better explore the mediating effect on motivations between human values and physical activity. We further test the separate mediation effects on autonomous motivation, controlled motivation and amotivation in single model.

**Mediation analysis on Relative Autonomous Index (RAI)**

First, test the mediation effect on RAI between values and physical activity. Gender, Age, BMI and Competition level were controlled in the model. The regression of the structural equation model demonstrated that the model was close to a good fit to the data, χ^2^/df=2.641, NFI=.887, IFI=.927, CFI=.924, RMSEA=.066. The factor loadings and path coefficients in the model are show in Fig S1.


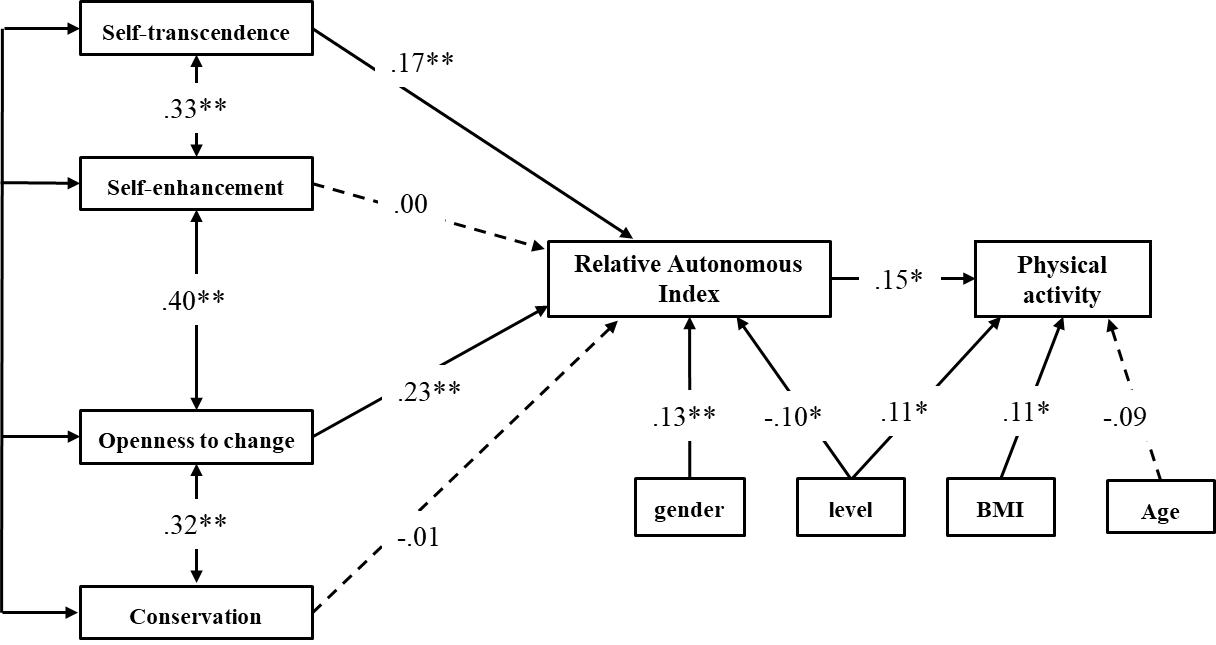


**Fig. S1. Mediation on relative autonomous index between the four higher order values and physical activity**

Under controlling of gender and competition level, Self-transcendence value (*B*=.17, *p*=.009, 95% CI [.05, .32]) and Openness to change (*B*=.23, *p*<.001, 95% CI [.12, .34]) positively predict RAI, whereas self-enhancement (*B*=-.00, *p*=.980, 95% CI [-.11, .11]) and conservation (*B*=-.01, *p*=.917, 95% CI [-.13, .12]) negatively predict RAI in the non-significant level.

Moreover, RAI positively predict physical activity (*B*=.15, *p*=.006, 95% CI [.07, .27]), under controlling of BMI (*B*=.10, *p*=.034, 95% CI [.01, .21]), competition level (*B*=.11, *p*=.033, 95% CI [.20, .22]) and age (*B*= -.09, *p*=.080, 95% CI [-.19, .01]).

The global model explains 4.8% variance of physical activity, *R^2^*=.048, *F*(9, 360)=3.131, *p*=.001. The indirect effect of the Self-transcendence value on physical activity through RAI are significant (*B*=.02, *SE*=.02, 95% CI [.04, .06], *p*=.012), due to the bias corrected 95% CI excludes zero. Similar, indirect effect of the openness to change value on physical activity through RAI are significant (*B*=.03, *SE*=.02, 95% CI [.01, .07], *p*=.006), due to the bias corrected 95% CI excludes zero (See in Table S4).

**Table S4. Indirect effect of the four higher order values on physical activity through motivations.**

| **Indirect Effect** | ***B*** | ***P*** | ***SE*** | Bootstrapping 95% CI | |
| --- | --- | --- | --- | --- | --- |
|  |  |  |  | **Lower** | **Upper** |
| **Model 1 : Mediation on relative autonomous index** | | | | | |
| Self-transcendence → RAI → PA | .02* | .012 | .02 | .00 | .06 |
| Self-enhancement → RAI → PA | .00 | .965 | .01 | -.02 | .02 |
| Openness to change → RAI → PA | .03** | .006 | .02 | .01 | .07 |
| Conservation → RAI → PA | -.00 | .845 | .01 | -.02 | .02 |
| **Model 2: Mediation on autonomous motivation** | | | | | |
| Self-transcendence → autonomous → PA | .02* | .027 | .02 | .00 | .06 |
| Self-enhancement → autonomous → PA | .01 | .217 | .01 | -.01 | .03 |
| Openness to change → autonomous → PA | .03* | .021 | .02 | .00 | .07 |
| Conservation→autonomous→PA | -.01 | .231 | .01 | -.03 | .01 |
| **Model 3: Mediation on controlled motivation** | | | | | |
| Self-transcendence → controlled → PA | -.00 | .465 | .01 | -.02 | .01 |
| Self-enhancement → controlled → PA | -.01 | .379 | .02 | -.05 | .02 |
| Openness to change → controlled → PA | .00 | .276 | .01 | -.00 | .03 |
| Conservation → controlled → PA | .01 | .282 | .01 | -.01 | .03 |
| **Model 4: Mediation on amotivation** | | | | | |
| Self-transcendence → amotivation → PA | .02* | .034 | .01 | .00 | .05 |
| Self-enhancement → amotivation → PA | .02* | .025 | .01 | .00 | .04 |
| Openness to change → amotivation → PA | .02* | .015 | .01 | .00 | .04 |
| Conservation → amotivation → PA | .00 | .947 | .01 | -.02 | .02 |

**Mediation effect on Autonomous motivation**

Next, test the mediation effect on autonomous motivation between values and physical activity. The suggested model was tested via structural regression analysis in Fig S2. Gender, BMI, age and Competition level were controlled in the model. The regression of the structural equation model demonstrated that the model was close to a good fit to the data, *χ*^2^/*df*=2.771, NFI=.882, IFI=.921, CFI=.918, RMSEA=.068.


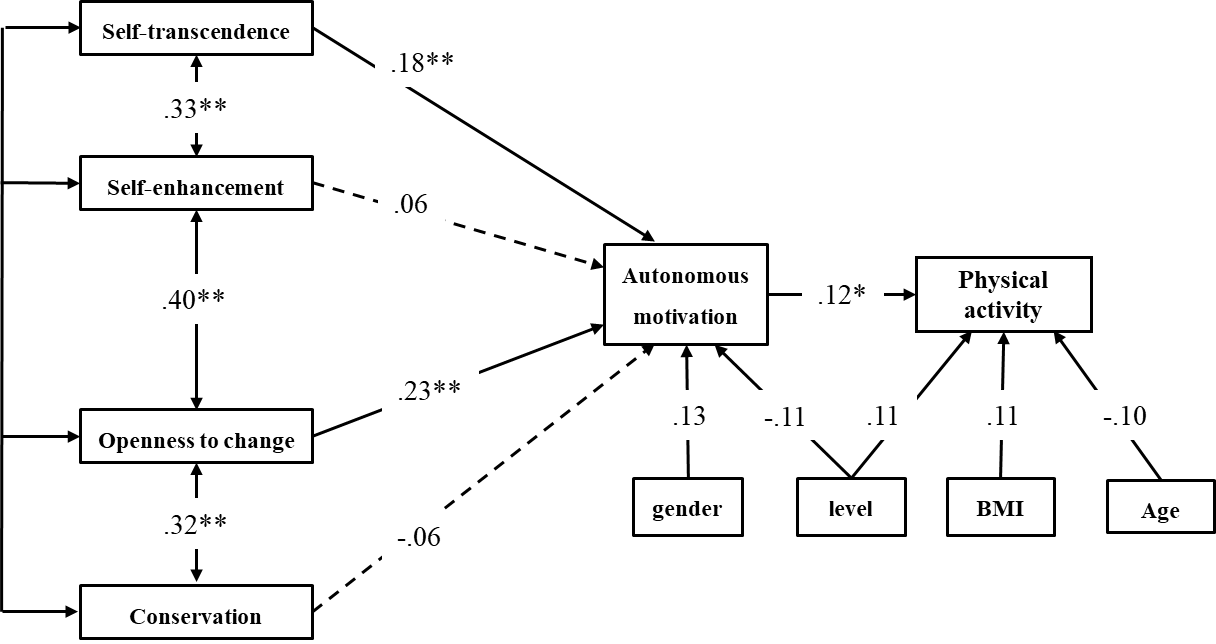


**Fig. S2. Mediation on autonomous motivation between the four higher order values and physical activiyty**

The factor loadings and path coefficients in the model are show in Fig S2. According to the results, Self-transcendence value (*B*=.18, *p*=.003, 95% CI [.05, .32]) and openness to change value (*B*=.23, *p*<.001, 95% CI [.13, .35]) positively predict autonomous motivation, after controlling gender (*B*=.24, *p*<.001, 95% CI [.15, .34]) and competition level (*B*=-.11, *p*=.020, 95% CI [-.20, -.02]), while Self-enhancement value (*B*=.06, *p*=.189, 95% CI [-.06, .17]) and Conservation value (*B*=-.06, *p*=.303, 95% CI [-.19, .07]) negatively predict autonomous motivation in the non-significantly.

Hence, autonomous motivation positively predicts global physical activity (*B*=.12, *p*=.023, 95% CI [.04, .22]), Openness to change positively predict global physical activity (*B*=.12, *p*=.036, 95% CI [.01, .22]), after controlling of BMI (*B*=.11, *p*=.030, 95% CI [.00, .20]) and competition level (*B*=.11, *p*=.034, 95% CI [.02, .22]).

The model explained 4.2% of the variance of global physical activity, *R*^2^=.042, *F*(9, 369)=2.84, *p*=.003. The indirect effect of the Self-transcendence value on physical activity through autonomous motivation are significant (*B*=.02, *SE*=.02, 95% CI [.00, .06], *p*=.027), due to the bias corrected 95% CI excludes zero. Similar, indirect effect of the openness to change value on physical activity through relative autonomous index are significant (*B*=.03, *SE*=.02, 95% CI [.00, .07], *p*=.021), due to the bias corrected 95% CI excludes zero (See in Table S4).

**Mediation effect on Controlled motivation**

The suggested model was tested via structural regression analysis. Gender, BMI and Competition level were controlled in the model. The regression of the structural equation model demonstrated that the model was close to a good fit to the data, *χ*^2^/*df*=2.868, NFI=.861, IFI=.905, CFI=.900, RMSEA=.07. The factor loadings and path coefficients in the model are show in Fig S3.


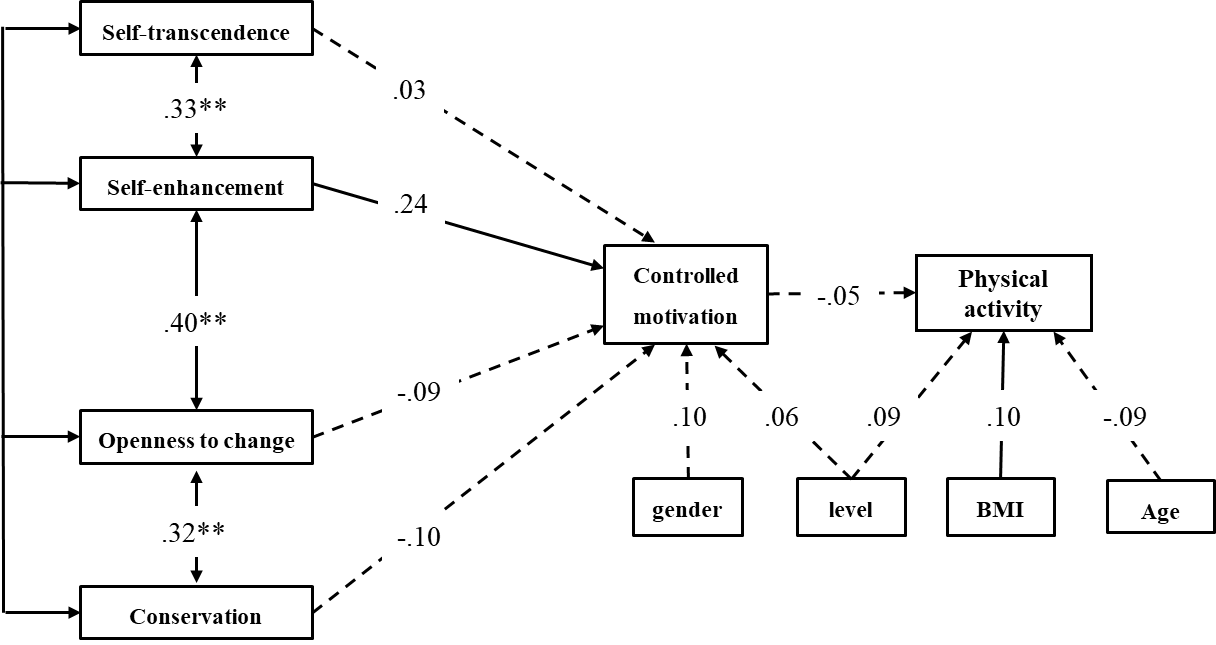


**Fig. S3. Mediation on controlled motivation between the four higher order values and physical activity**

After controlling of gender (*B*=.10, *p*=.061, 95% CI [-.10, .20]) and competition level (*B*=.06, *p*=.238, 95% CI [-.04, .16]), Self-enhancement values (*B*=.24, *p*<.001, 95% CI [.12, .35]) positively predict controlled motivation, other values do not predict controlled motivation significantly. Moreover, controlled motivation (*B*=-.05, *p*=.355, 95% CI [-.15, .06]) negatively predicts physical activity in the non-significant level. Indirect effect of four higher order values on global physical activity through controlled motivation are not significant, due to the bias corrected 95% CI includes zero (See in Table S4).

**Mediation effect on Amotivation**

The suggested model was tested via structural regression analysis. Gender, Age, BMI, and Competition level were controlled in the model. The regression of the structural equation model demonstrated that the model was close to a good fit to the data, *χ*^2^/*df*=2.922, NFI=.862, IFI=.905, CFI=.901, RMSEA=.071. The factor loadings and path coefficients in the model are show in Fig S4.


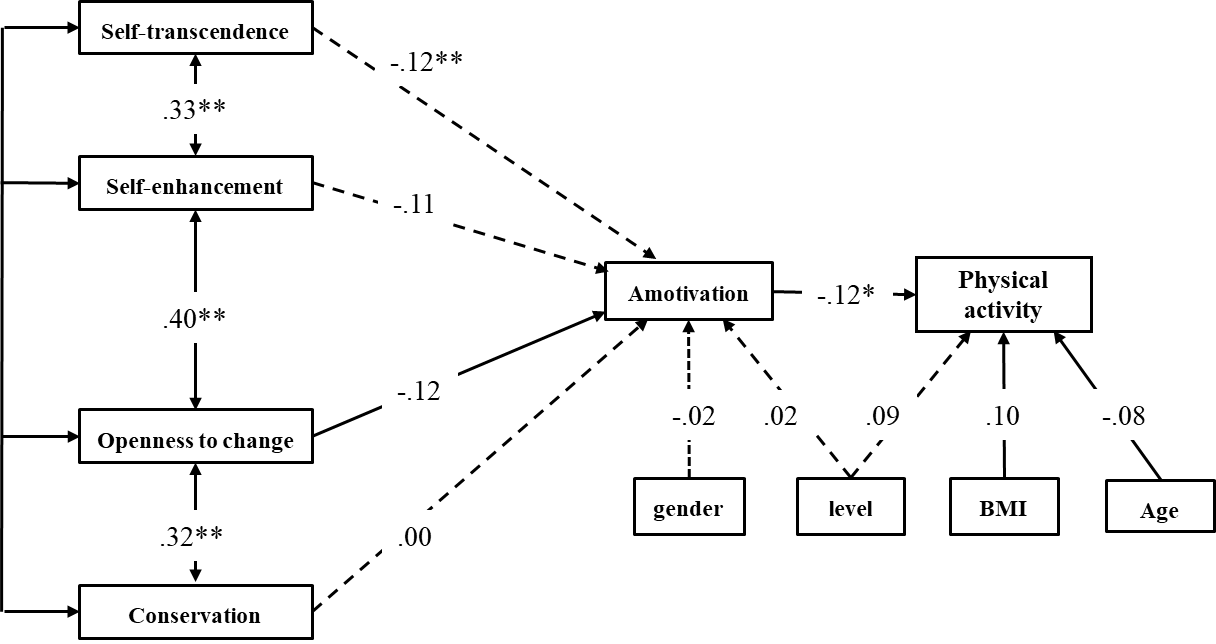


**Fig. S4. Mediation on amotivation between the four higher order values and physical activiy**

Openness to change negatively predicts amotivation (*B*=-.12, *p*=.039, 95% CI [-.22, .01]), Self-transcendence value (*B*=-.13, *p*=.060, 95% CI [-.26, .01]) and self-enhancement value (*B*=-.11, *p*=.061, 95% CI [-.22, .01]) marginally predict controlled motivation, Conservation insignificantly predict amotivation (*B*=.00, *p*=.970, 95% CI [-.13, .13]). Moreover, amotivation negatively predicts physical activity (*B*=-.14, *p*=.007, 95% CI [.26, -.06]).

The model explains 4.9% variance of physical activity, *R^2^*=.05, *F*(9, 369)=3.149, *p*=.001. The indirect effect of the Self-transcendence value on physical activity through amotivation are significant (*B*=.02, *SE*=.01, *p*=.034, 95% CI [.00, .05]), due to the bias corrected 95% CI excludes zero. Similarly, indirect effect of the Self-enhancement value on physical activity through amotivation are significant (*B*=.02, *SE*=.01, *p*=.025, 95% CI [.02, .04]), due to the bias corrected 95% CI excludes zero. Last, indirect effect of the Openness to change value on physical activity through amotivation are significant (*B*=.02, *SE*=.01, *p*=.015, 95% CI [.00, .04]), due to the bias corrected 95% CI excludes zero (See in Table S4).

**Reference**

1. Macfarlane DJ, Lee CCY, Ho EYK, Chan KL, Chan DTS. Reliability and validity of the Chinese version of IPAQ (short, last 7 days). J Sci Med Sport. 2007; 10(1): 45–51. <https://doi.org/10.1016/j.jsams.2006.05.003> PMID: 16807105
2. Gao ZH, Yang SQ, Margraf J, Zhang XC, Lu P. Reliability and Validity Test for Schwartz’s Portrait Values Questionnaire (PVQ-21) in Chinese College Students. Chin J Health Psychol, 2016; 24(11): 1684-1688.
3. Davidov E, Schmidt P, Schwartz SH. Bringing Values Back In: The Adequacy of the European Social Survey to Measure Values in 20 Countries. Public Opin Q. 2008; 72(3):420–445. <https://doi.org/10.1093/poq/nfn035>
4. Markland D, Tobin V. A modification to the Behavioural Regulation in Exercise Questionnaire to include an assessment of amotivation. J Sport Exerc Psychol. 2004; 26(2): 191–196. <https://doi.org/10.1123/jsep.26.2.191>
5. Hu LT, Bentler PM. Cutoff criteria for fit indexes in covariance structure analysis: Conventional criteria versus new alternatives. Struct Equ Modeling. 1999; 6(1): 1–55. <https://doi.org/1.1080/10705519909540118>
6. Hayes, A. F. Introduction to mediation, moderation, and conditional process analysis: A regression-based approach (3rd edition). New York: The Guilford Press, 2002.
7. IBM Corp. Released IBM SPSS Statistics for Windows, Version 2. Armonk, NY: IBM Corp, 2012.
